# An Integrative Review of the Influence on Human Papillomavirus Vaccination Adherence among Adolescents

**DOI:** 10.3390/healthcare11182534

**Published:** 2023-09-13

**Authors:** Hyewon Shin, Sunyeob Choi, Ju-Young Lee

**Affiliations:** 1 College of Nursing, Ewha Womans University, 52, Ewhayeodae-gil, Seodaemun-gu, Seoul 03760, Republic of Korea; hyeshin@ewha.ac.kr (H.S.); csy9309@ewha.ac.kr (S.C.); 2 College of Nursing, The Catholic University of Korea, 222 Banpo-daero, Seocho-gu, Seoul 06591, Republic of Korea

**Keywords:** human papillomavirus viruses, vaccination, intention

## Abstract

To enhance the vaccination rate of adolescents against HPV, understanding their current vaccination intentions and identifying the factors that influence their decision to vaccinate are imperative. The Preferred Reporting Items for Systematic Reviews and Meta-Analysis (PRISMA) was used to search for relative literature. Five electronic databases (CINAHL, PubMed, Web of science, Research Information Sharing Service (RISS) and Korea Citation Index (KCI)) were searched from 2007 to 2022, and 19 studies were selected for final review. The Mixed Methods Assessment Tool (MMAT) was used as a critical appraisal tool to evaluate the quality for various types of research designs. This integrative review revealed three themes, including facilitators (personal characteristics and environmental factors), barriers (lack of awareness and knowledge about HPV, concerns about side effects, and the burden of costs), and ways to increase vaccination uptake (knowledge promotion and HPV vaccines coverage by public insurance). To encourage HPV vaccine uptake, it is essential to enhance public knowledge, create compelling advertisements, and ensure that the vaccines are covered by public insurance. Moreover, providing educational programs that emphasize the effectiveness and significance of HPV vaccines to adults who can influence the vaccination decisions of adolescents is vital.

## 1. Introduction

Human papillomavirus (HPV) is the most common sexually transmitted infection in the world, and it is estimated that over 80% of women and 90% of men will be infected with the virus during their lifetime [1]). HPV has been recognized as the leading cause of cervical cancer, which serves as the fourth-most prevalent cancer among women worldwide [2]. For males, HPV has a significant causal role, which is associated with anogenital warts, precancerous lesions, and cancer such as anal and oropharyngeal. Fortunately, most of these diseases can be prevented through HPV vaccination. Therefore, the importance of HPV vaccination for both men and women is emphasized.

Vaccination is an essential tool to prevent and control HPV infection and related complications; the Advisory Committee on Immunization Practices (ACIP) recommends HPV vaccination between the ages of 9 and 26, with a focus on ages between 11 and 12 [3]. The ACIP has recommended HPV vaccination for female adolescents since 2006 and for male adolescents since 2009 [3]. As of June 2020, approximately 55% (107 out of 194) of the World Health Organization (WHO) Member States have successfully implemented HPV vaccination programs. However, the global population coverage for the final dose of HPV vaccination in 2019 was only 15%, significantly below the target of achieving 90% coverage by 2030 [4]. In 2016, Korea has also included HPV in a national vaccination program for only school-age girls (ages 12 to 13). Since the introduction of free vaccination, the HPV vaccination status of female adolescents, who were eligible for vaccination, has been rising in small increments every year [5]. Even so, the female adolescents’ vaccination rate of 16.9% is the lowest compared to other age groups [6]. Moreover, the HPV vaccination was not included in the mandatory national vaccinations for men and did not extend to all adult women. The rate for male adolescents is reported at 1.6% in South Korea [5], which is very low compared to 56.5% in the United States [7]. Korean mothers typically possess limited knowledge regarding the vaccination of boys against HPV, tend to underestimate the associated risks, and frequently exhibit hesitancy in administering the vaccine to their sons [8].

In order to increase the vaccination of adolescents, it will be essential to understand the current vaccination intention and to identify facilitators and barriers to provide an overview for key stakeholders. The underlying variables that impact individuals’ intentions to vaccinate should be identified, as these intentions act as a precursor to actual vaccination behavior. It is anticipated that individuals would possess the intention to modify behaviors related to health before engaging in indicated behaviors [9]. In many countries, studies have been conducted to identify the factors that influence vaccination intention, including at the personal, family, and health care provider levels. As HPV-related knowledge and positive attitudes towards HPV vaccination increased, the HPV vaccination rates increased significantly [10,11]. In another study, psychosocial factors such as parental knowledge of HPV and its safety, perceived benefit, perceived susceptibility, perceived vaccine efficacy, and information availability were identified as influencing factors.

At the health provider level, factors that promote vaccination include the provision of vaccination information [12,13,14,15,16]. Concerns regarding the safety of vaccination, lack of knowledge about the need for vaccination [10,11], and fear of side effects and financial reasons are barriers to the intention to immunize [17]. In a study of the factors influencing HPV vaccination intention in Korean adolescents, vaccine-related knowledge and positive attitudes towards the vaccine (i.e., belief in the safety and efficacy of the vaccine) were among the personal cognitive factors associated with vaccine uptake [12].

Given that vaccination intention is the strongest predictor of HPV vaccination [18], understanding the facilitators and barriers can be used to guide appropriate promotion to increase vaccination rate. To date, the existing systematic reviews have summarized predictors of HPV vaccine intention and decision-making in various countries; however, based on our knowledge, no study has investigated the multidimensional influencing factors and intention to receive HPV vaccination among adolescents in Korea. This gap in the existing literature needs to be addressed. Therefore, this integrative review aims to identify the factors that influence female and male HPV vaccination intention considering personal, parent, psychosocial, and environmental factors.

Specifically, this integrative review presents (1) the factors associated with the HPV vaccination among Korean adolescents, (2) the knowledge, intention, vaccination rate of the HPV vaccination among Korean adolescents, and (3) the facilitators and barriers of HPV vaccination among Korean adolescents. Understanding these factors would provide healthcare providers and stakeholders with the essential knowledge necessary to develop strategies that may significantly enhance HPV vaccine uptake among Korean adolescents.

## 2. Methods

### 2.1. Search Strategy

The Preferred Reporting Items for Systematic Reviews and Meta-Analysis (PRISMA) [19] was used to search for related literature. The PRISMA guidelines were developed with the objective of enhancing the clarity, transparency, and consistency of reporting in systematic reviews and meta-analyses [19]. To search for eligible studies, we used five databases: Cumulative Index to Nursing Allied Health Literature (CINAHL), PubMed, Web of science, and two Korean databases including Research Information Sharing Service (RISS) and Korea Citation Index (KCI), from 2007 to December 2022. The selection of these databases was based on their relevance to the topic, and multiple databases were utilized to reduce potential biases. The search was conducted by three researchers (SH, LJ, CS) independently. The search terms were constructed using truncation and Boolean operators. The specific search terms were as follows: (children or adolescents or youth or child or teenager) AND (hpv vaccine or hpv vaccination or human papillomavirus vaccine) AND (intention or motivation or uptake).

A research librarian helped to find additional articles. Boolean operators and truncation were used with combinations of the keywords in order to identify all relevant articles. The librarians suggested search terms and databases based on the researchers’ study topics. Subsequently, both the researchers and the librarians conducted individual searches. The librarians provided references that the researchers might have missed.

The article inclusion criteria were as follows: (1) original studies published in English/Korean language, peer-reviewed journals; (2) studies of participants that were school-aged children aged 8 to 18 in the Korean education system, parents or caregivers of school-aged children and teachers of school-aged children who may influence the vaccination of the student; and (3) papers that identify the purpose of HPV vaccination and the relative factors; knowledge related to HPV vaccination, attitudes toward the HPV vaccination, HPV vaccination behaviors, and HPV vaccination intention. Systematic/literature reviews, opinion pieces, editorials, conference proceedings and studies published in languages other than English and Korean were excluded.

**Figure 1:**
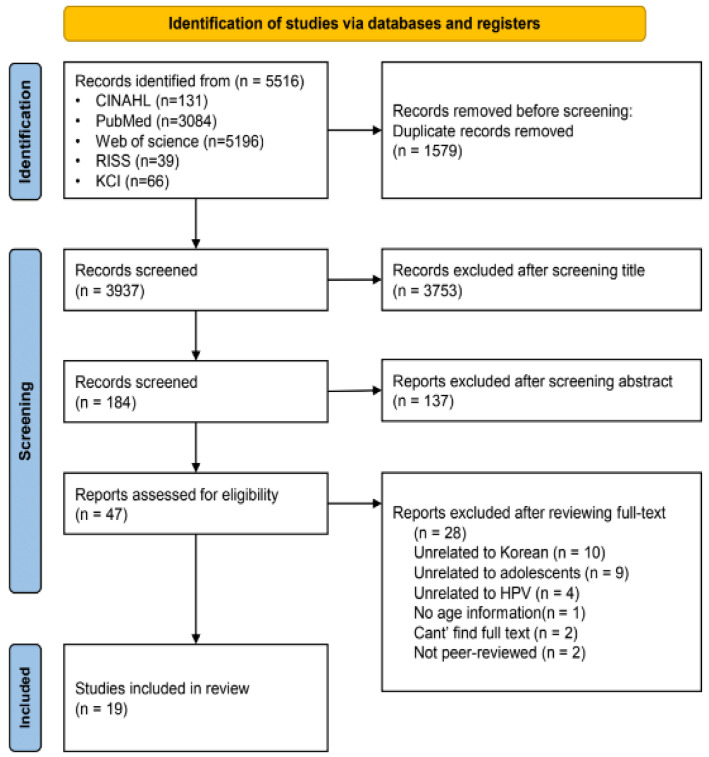
PRISMA flow diagram.

A PRISMA flowchart [19] was used to select the relative literature (Figure 1). A total of 5516 studies were searched from five databases: CINAHL (n = 131), PubMed (n = 3084), Web of Science (n = 2196), RISS (n = 39) and KCI (n = 66). A total of 1579 duplicates were removed and 3753 articles were eliminated after screening the title adhering to the inclusion criteria of the study. This process ensured that papers not pertinent to the study’s scope, such as those not targeting adolescents, unrelated to HPV vaccines, or not associated with HPV vaccination, were excluded. An additional 137 articles were excluded after screening the abstract. The purpose of title and abstract screenings is to eliminate articles that are not relevant to the topic. A total of 47 articles were selected for full-text review. A total of 28 articles were excluded for several reasons including being unrelated to adolescents (n = 10), unrelated to Korean populations (n = 8), unrelated to HPV (n = 4), not containing the age information of participants (n = 1), not being a peer-reviewed paper (n = 4), and being a review paper (n = 1). The final 19 articles were selected for review. The full details are shown in Table 1.

### 2.2. Quality Appraisal

We assessed the risk of bias of each study using the MMAT in Table 2. These tools rated the quality of the methodology used to know whether the research approach was appropriate to answer the research question, the findings were adequately derived from the data, the correctness of the interpretation of results, the representativeness of the target population, and the appropriateness of the measurements and statistical analysis. Content analysis is an appropriate research method for analyzing compiled research data to build new knowledge and generate theories. It enables the classification and examination of research paper content without influencing existing data. However, it relies solely on recorded data and has limitations due to subjective researcher involvement in securing practical validity [20]. Three researchers (SH, LJ, CS) appraised the quality of the selected articles using the Mixed Methods Assessment Tool (MMAT) [21] for each research design. The MMAT was the critical appraisal tool to appraise the quality for various studies, including quantitative, qualitative and mixed methods studies [21]. First, the three researchers independently determined the appropriateness of inclusion according to the screening questions; clarification of research questions and the appropriateness of the research question and data collection method. Then, the researchers classified the categories of each article according to the research method, and evaluated each article based on MMAT Methodological Quality Criteria. As the selected articles were qualitative and quantitative studies, we used MMAT Methodological Quality Criteria for quantitative descriptive studies and non-randomized studies. The researchers respond to each category using ‘yes’, ‘no’, and ‘cannot tell’. The rate of agreement was 98% (4 items out of 200 had disagreement). The items of disagreement were related to the “risk of nonresponse bias” and the “appropriateness of measurements”. Three researchers discussed these items. Regarding the “risk of nonresponse bias”, there was no description of the questionnaire administration and retrieval, but the final number of participants was stated. The researchers reached a consensus of “cannot tell”. Regarding the “appropriateness of measurements”, since there was no presentation of the reliability and validity of the measurement tools, and the consensus was “no”. Next, the three researchers discussed the disagreement and reached a consensus. No article was excluded after quality evaluation according to MMAT guidelines, because it might have caused bias [22]. Two papers were scored as low [23,24], four papers as moderate [25,26,27,28], and thirteen as very high [10,12,29,30,31,32,33,34,35,36,37,38,39]. Two papers with low scores were retained because despite having participants from limited areas, they investigated important ideas related to this systematic review.

### 2.3. Data Extraction

The data extraction form was rigorously tested using two articles to ensure that the form captured all relevant information. Primary data from all articles were extracted by two researchers (SH and CS), and organized into an Excel table. The extracted data included the author and publication year, methodology, sample, purpose, the findings, and the strengths and limitations. In instances of disagreement, a discussion was conducted with the participation of LJ in order to reach a consensus.

### 2.4. Data Synthesis

Following the completion of the data extraction process, the obtained findings were subjected to analysis and subsequently organized into distinct thematic areas using a thematic analysis approach. We followed Thomas and Harden’s three stages of thematic analysis, namely, coding, organizing codes into descriptive themes, and integrating descriptive themes into analytical themes [40]. In order to obtain a comprehensive understanding of the literature, the selected articles were thoroughly examined and reviewed multiple times. The analysis of the articles was conducted by two researchers (SH and CS), with each researcher responsible for analyzing half of the articles. The results obtained from the analyses were subsequently reviewed and refined by the entire research team.

**Table 1:** Summary of evidence of HPV studies (n = 19).

Author (Year)	Methods	Sample	Purpose	Findings (Vaccination—a. Rate, b. Intention, c. Knowledge, d. Attitude, e. Others) (n/%/Mean)	Findings (Independent/Dependent Variable)	Strengths/ Limitations	Quality Score
Choi & Park (2016) [25]	QT: Cross-sectional descriptive study (Self-reporting questionnaire)	n = 495 male students (high school and university students)	To survey the current state of HPV vaccination and the predictors of vaccination intention among Korean male students of high school (ages 15–19) and university (ages 17–27).	a. very low. 2.4% (ex. 12 students out of 495) c. low. 11.76 out of 100 e. overall health belief: low. 2.18 out of 4	The significant predictors promoting the intention of HPV vaccination were university students, the experience of sexual intercourse, and perceiving the benefit of HPV vaccination.	S: Use a theoretical framework L: Cross-sectional study, convenience sampling	80%
Choi & Cheon (2015) [26]	QT: Cross-sectional descriptive study (Self-reporting questionnaire)	n = 492 female adolescents (middle and high school)	To assess HPV vaccination coverage, intention to HPV vaccination, cervical cancer knowledge, and HPV knowledge among female middle and high school students.	a. low. 10.2% b. 54.2% c. 62.2% e. cervical cancer knowledge: 58.6%	The subjects had moderate knowledge of cervical cancer and HPV. There were significant differences in cervical cancer knowledge scores between the intended group and the not-intended group for vaccination.	L: Cross-sectional study, data collection was conducted in specific areas	80%
Han & Son (2021) [29]	QT: Cross-sectional descriptive study (Self-reporting questionnaire)	n = 230 mothers of daughters of middle school (2nd grade)	To investigate environmental factors influencing mothers’ decision-making regarding vaccination of HPV for their daughters.	a. 75.7% (The rate of the daughters) b. 53.6% c. 9.79 out of 23 d. 21.27 out of 63	The factors affecting HPV vaccination intention were attitudes toward HPV vaccination, information communicated by healthcare providers, and the provision of positive information regarding the HPV vaccination.	S: Proportional sampling considering the sizes of the metropolises and provinces L: Cross-sectional study, data collection was conducted in specific areas	100%
Hong (2019) [30]	QT: Cross-sectional descriptive study (Self-reporting questionnaire)	n = 249 mothers of daughters in elementary and middle school	To identify factors that influence HPV vaccination intention and behavior for mothers with a teenage daughter as the subject of HPV vaccine-free inoculation from 2016 based on the theory of planned behavior.	a. 41.8% (The rate of daughters) b. 18.84/14.63 (vaccinated/unvaccinated) out of 21 d. 18.89/15.34 out of 21 e. subjective norms: 17.76/14.55 out of 21, perceived behavior control: 25.06/21.88 out of 28 b, d, e: showed a significant difference between the subjects HPV vaccination status	The factors affecting the HPV vaccination intention were attitude, subjective norm, and perceived behavior control in order. The factors influencing HPV vaccination behavior were level of education, subject’s vaccination status, recommendation of health care provider, vaccination status of surrounding people, intention, etc.	S: Use a theoretical framework L: Cross-sectional study, convenience sampling, subjects mostly residing in rural areas	100%
Hong & Chung (2019) [31]	QT: Cross-sectional descriptive study (Self-reporting questionnaire)	n = 285 mothers of adolescent daughters (12–13 years)	To evaluate the accuracy of an HPV vaccination behavior model based on the health belief model.	a. high. 42.1% (The rate of daughters) b. high. 5.67 out of 7 e. vaccination-related health belief: perceived susceptibility: 2.04 out of 4, perceived seriousness: 2.90 out of 4, perceived benefit: 3.09 out of 4, perceived barrier: 2.09 out of 4 The vaccination rate was high due to the change in the perception of the parents and the introduction of the national vaccination program. Perceived benefits had effects on vaccination behavior by completely mediating the intention.	The vaccination intention was an important variable for predicting vaccination behaviors. Perceived benefits had effects on the vaccinating behavior by completely mediating the intention, while the perceived barriers had effects on the vaccinating behavior by partially mediating the intention.	S: Use a theoretical framework L: Cross-sectional study, convenience sampling, subjects mostly residing in rural areas	100%
Jang (2018) [23]	QT: Cross-sectional descriptive study (Self-reporting questionnaire)	n = 246 male, n = 298 female students in high school	To identify and compare the factors associated with HPV vaccination intention between male and female high school students	a. 1.2%/16.4% (male/female): very low b. 47.6% (male)/86.2% (female) c. very low. 2.33 (male)/3.99 (F) out of 13~14 d. 3.07 (male)/3.31 (female) out of 5 e. vaccination-related health belief: 2.36/2.37 out of 4	The factors affecting the HPV vaccination of male students were religion, sexual experience, safety concerns, perceived needs, the importance of prevention, and perceived susceptibility. The factors affecting the HPV vaccination of female students were awareness of HPV vaccination and the importance of prevention.	S: Use a theoretical framework L: Cross-sectional study, convenience sampling, convenience sampling from a single site.	60%
Kang & Lee (2021) [10]	QT: Cross-sectional descriptive study (Self-reporting questionnaire)	n = 231 parents of sons in elementary school (5–6th grades)	To determine how attitude, subjective norm, and perceived behavioral control (PBC) were related to parents’ intentions to vaccinate their sons in elementary school against HPV, applying the updated theory of planned behavior.	b. 13.87 out of 21 d. 15.46 out of 21 e. subjective norms: 11.45 out of 21, perceived behavior: 20.52 out of 28 * e directly predicted the b	The simple effect of attitude to intention was significant under three different levels of the PBC (low, moderate, and high), but the magnitudes of the relationships were not homogeneous. Subjective norms and PBC directly predicted the intention of HPV vaccination.	S: Use a theoretical framework L: Cross-sectional study, convenience sampling	100%
Kang & Moneyham (2011) [32]	QT: Cross-sectional descriptive study (Self-reporting questionnaire)	n = 726 high school girls, n = 667 mothers	To examine the attitudes, intentions, and perceived barriers to HPV vaccination among Korean high school girls and their mothers.	a. 0.8% (the rate of high school girls) b. 3.32/3.43 (girl/mother) out of 5 d. favorable 2.98/2.98 (girl/mother) out of 5	The major barriers to HPV vaccination were the high cost, the fact that not many people they knew had received the vaccination, and the HPV vaccination had not been personally recommended.	S: Samples were collected in various region L: Cross-sectional study	100%
Kang (2011) [33]	QT: Cross-sectional descriptive study (Self-reporting questionnaire)	n = 101 mothers of adolescent girls	To examine mothers’ knowledge about HPV vaccination to prevent cervical cancer in Korea.	a. 5.9% (the rate of the daughters) c. 24.2 out of 100 (low) * c of the vaccination group was significantly higher than that of the non-vaccinated group	The HPV vaccine knowledge score of the vaccination group was significantly higher than that of the non-vaccinated group. The reasons for not vaccinating their daughters against the HPV were the financial burden, the lack of HPV knowledge, and worries about possible side effects.	L: Cross-sectional study, convenience sampling from a single site.	100%
Kim (2012) [34]	QT: Cross-sectional descriptive design (Self-reporting questionnaire)	n = 757 health teachers of elementary, middle, high, and special schools	To assess HPV knowledge, compare the health beliefs toward HPV vaccination and intention to recommend HPV vaccination for girls and boys, and identify the factors influencing the intention to recommend HPV vaccination for girls and boys among Korean health teachers.	c. 7.71 out of 20 (low) b. 4.60/5.80 (boys/girls): Intention to recommend HPV vaccination e. HPV awareness: 76.4% There are differences in their intention to recommend HPV vaccine for girls and boys.	Factors related to the intention of vaccination for girls were the HPV vaccination status of the health teachers’ children, the teachers’ Pap-test experience, perceived benefits, susceptibility, and barriers. -Factors related to the intention to HPV vaccination for boys were the health teachers’ career duration, HPV knowledge, perceived benefits, susceptibility, and severity.	S: A highly representative sample was used, using a theoretical framework L: (1) Cross-sectional study, (2) Convenience sampling	100%
Kim (2015) [27]	QT: Quasi-experimental study	n = 117 of students in elementary school (5th grade)	To determine the awareness among fifth-grade girls and boys of sexually transmitted diseases (STDs), cancer, and HPV, and to determine the factors associated with intention to obtain the HPV vaccination.	b. 4.17/4.26 (boys/girls) out of 5 after education c. 4.58/4.95 (boys/girls) out of 8 after education * After education, there was no gender difference in b and c (no pre-test done)	There were significant gender differences with respect to responses to the statements “STD is preventable” and “cancer is preventable”, and concerns about the pain associated with vaccine injection. After HPV education, there were no significant gender differences in HPV knowledge and intention to obtain the HPV vaccination.	S: Experimental study design L: Lack of a control group, convenience sampling from a single site.	60%
Kim & Choi (2017) [28]	QT: Cross-sectional descriptive study (Self-reporting questionnaire)	n = 200 mothers of teenagers who were not vaccinated against HPV	To examine the intention of mothers to vaccinate their teenage children against HPV infection, according to the children’s sex. Based on the theory of planned behavior, the study identified the sex-specific predictors of mothers’ intention to vaccinate their teenage children against HPV.	b. 4.59/4.94 (boys/girls) d. 4.98/5.39 (boys/girls) e. subjective norms: 4.31/4.62, perceived behavior control: 5.31/5.31 * The differences between b, d, & e scores were not statistically significant between their children’s sex.	Among the mother with sons, the intention to vaccinate against HPV was significantly correlated with attitude, PBC, and subjective norms. Among the mothers with daughters, the intention to vaccinate against HPV also was significantly correlated with attitude, PBC, and subjective norms.	S: Use a theoretical framework L: Cross-sectional study, convenience sampling from a single site.	100%
Kim et al. (2019) [35]	QT: Cross-sectional descriptive study (Self-reporting questionnaire)	n = 121 mothers of daughters in elementary school	To identify the impacts of HPV vaccination-related health beliefs, attitudes toward HPV vaccination, and subjective norms on HPV vaccination intent targeting mothers of elementary school daughters.	a. 11.6% (The rate of the daughters) b. 17.35 out of 21 d. 17.96 out of 21 e. vaccination-related health belief: 30.45 out of 44, Subjective norms: 16.26 out of 21 Attitudes toward HPV vaccination, subjective norms, vaccination plans for their children, and mother’s vaccination status were significant factors influencing HPV vaccination intention. These factors accounted for 72% of the HPV vaccination intention.	The factors affecting HPV vaccination intention were attitude toward HPV vaccination, subjective norms, vaccination plans for their children, and the mother’s vaccination status. The biggest influencing factor was HPV vaccination attitudes.	S: Use a theoretical framework L: Cross-sectional study, data collection from a limited regional area	100%
Lee et al. (2017) [12]	QT: Cross-sectional descriptive design (Self-reporting questionnaire)	n = 140 mothers of daughters in elementary and middle schools (9~14 years old)	To assess the ranges of perceptions and personal experience and their influences on attitudes regarding HPV vaccinations of children among mothers of adolescent daughters.	b. 70% c. 48% The more the participants’ knowledge about HPV infection and about the relationship of HPV to cervical cancer, the more positive their attitudes.	The more the participants’ pre-knowledge about HPV infection and about the relationship of HPV to cervical cancer, the more positive their attitudes. As the level of education rose, the proportion of mothers with negative attitudes toward vaccinating their adolescent daughters rose as well.	S: The questionnaire was distributed randomly in three different schools L: Cross-section study, small sample size, and questionnaire were distributed in a well-developed city in Korea	100%
Nam & Lee (2021) [24]	QT: Cross-sectional comparative design (Self-reporting questionnaire)	n = 171 mothers of daughters in elementary school (5–6th grade)	To provide basic data for the development of a nursing intervention program in order to improve the intention to receive HPV vaccination intention of mothers of daughters in elementary school by confirming the mediating effect of self-efficacy in the relationship between knowledge of HPV vaccine, attitude towards vaccination, subjective norm, and intention to receive a vaccination.	a. 15.2% (The rate of the daughters) b. high. 28.38 out of 35 c. average. 7.91 out of 16 d. high. 22.57 out of 28 e. subjective norms: high. 5.75 out of −12 to 12, self-efficacy: high. 16.65 out of 21 The fully mediating effect of self-efficacy was confirmed in the relationship between the knowledge of the HPV vaccine and the intention to receive a vaccination.	The fully mediating effect of self-efficacy in the relationship between the knowledge of the HPV vaccine and the intention of the vaccinations. The partial mediating effect of self-efficacy was confirmed in the relationship between attitude towards HPV vaccination and the intention of vaccination. The partial mediating effect of self-efficacy was confirmed in the relationship between the subjective norm for HPV vaccination and the intention to receive a vaccination.	S: Use a theoretical framework L: Cross-sectional study, data collection from limited regional area	60%
Oh & Lee (2018) [36]	QT: Cross-sectional descriptive study (Self-reporting questionnaire)	n = 132 mothers of students in elementary school	To identify the factors associated with the intention of HPV vaccination among mothers of daughters in elementary school.	a. 5.3% (The rate of the daughters) b. 8.67 out of 21 c. 7.55 out of 16 d. 7.42 out of 21 e. subjective norms: 7.09 out of 21, perceived behavior control: 15.77 out of 35 The attitudes toward vaccination, perceived behavior and subjective norms were significant factors influencing HPV vaccination intention.	The factors affecting the predictor of vaccination were attitude toward HPV vaccination, perceived behavior control, and subjective norm as meaningful predictors.	S: Use a theoretical framework L: Cross-sectional study, data collection from the limited regional area	100%
Park & Kim (2020) [37]	QT: Cross-sectional descriptive study (Self-reporting questionnaire)	n = 151 mothers of boys in elementary school (4–6th grade)	To identify the factors influencing mothers’ intention to vaccinate their elementary-school sons against HPV.	a. none. 0% (The rate of the sons) b. 5.04 out of 7 c. 11.13 out of 20 d. 5.25 out of 7 e. subjective norms: 4.47 out of 7, self-efficacy: 4.94 out of 7 Self-efficacy and subjective norms towards HPV vaccination were significant factors influencing mothers’ intention to vaccinate their elementary-school sons.	The factors that influenced the intention of vaccination are self-efficacy and subjective norms towards HPV vaccination.	S: Use a theoretical framework L: Cross-sectional study, data collection from a limited regional area, response bias by using a self-report questionnaire	100%
Park & Jang (2017) [38]	QT: Cross-sectional descriptive study (Self-reporting questionnaire)	n = 298 mothers of daughters in middle or high school	To identify the factors that influence the practices and the intentions of HPV vaccination among adolescent daughters’ mothers.	a. 13.1% (The rate of the daughters) b. 84.6% c. 5.8 out of 15 e. sex-related communication: 79.9%(sex), 72.8%(STD), 73.2%(contraception) The intention to receive HPV vaccination was mainly influenced by HPV knowledge.	The factors that influenced HPV vaccination most were their family history of cervical cancer, educational backgrounds, and awareness of the HPV vaccine.	L: Cross-sectional study, convenience sampling, data collection from the limited regional area	100%
Yoo (2014) [39]	QT: Cross-sectional descriptive study (Self-reporting questionnaire)	n = 234 mothers of daughters in high school	To examine the knowledge level of HPV, cervical cancer, and vaccination status among Korean mothers of daughters in high school.	a. 3.9% (the rate of the daughters) low b. 85% (high) c. 4.21 out of 20 e. HPV knowledge: 3.88 out of 7	The major barrier to HPV vaccination was the concern for side effects from the vaccination. The most effective recommendation for HPV vaccination came from healthcare providers.	L: Cross-sectional study, data collection from a limited regional area, the proportion of low-income and low-educated people among the subjects is relatively low	100%

* Note: Authors assigned a number to each article based on American Psychological Association (APA) formatting. Abbreviations; QT, quantitative design; L, limitations of evidence; S, strengths Quality Appraisal: We appraised articles using the Mixed Methods Appraisal Tool (MMAT) [21]. An article could score 0, 25, 50, 75, or 100% based on how many assessment items the article addressed. For individual quality scores, see Table 2.

**Table 2:** Risk-of-bias assessment using MMAT.

Study Number #	1	2	3	4	5	6	7	8	9	10	11	12	13	14	15	16	17	18	19
Content	Choi & Park (2016) [25]	Choi & Cheon (2015) [26]	Han & Son (2017) [29]	Hong (2019) [30]	Hong & Chung (2019) [31]	Jang (2018) [23]	Kang & Lee (2021) [10]	Kang & Moneyham (2011) [32]	Kang (2011) [33]	Kim (2012) [34]	Kim (2015) [27]	Kim & Choi (2017) [28]	Kim et al. (2019) [35]	Lee et al. (2017) [12]	Nam & Lee (2021) [20]	Oh & Lee (2018) [36]	Park & Kim (2020) [37]	Park & Jang (2017) [38]	Yoo (2014) [39]
**Q1. Qualitative Studies**																			
Is the qualitative approach appropriate to answer the research question?																			
Are the qualitative data collection methods adequate to address the research question?																			
Are the findings adequately derived from the data?																			
Is the interpretation of results sufficiently substantiated by data?																			
Is there coherence between qualitative data sources, collection, analysis and interpretation?																			
**Q3. Non-Randomized Studies**																			
Are the participants representative of the target population?											N								
Are measurements appropriate regarding both the outcome and intervention (or exposure)?											Y								
Are there complete outcome data?											Y								
Are the confounders accounted for in the design and analysis?											N								
During the study period, is the intervention administered (or exposure occurred) as intended?											Y								
**Q4. Quantitative Descriptive Studies**																			
Is the sampling strategy relevant to address the research question?	Y	Y	Y	Y	Y	Y	Y	Y	Y	Y		Y	Y	Y	Y	Y	Y	Y	Y
Is the sample representative of the target population?	N	N	Y	Y	Y	N	Y	Y	Y	Y		Y	Y	Y	Y	Y	Y	Y	Y
Are the measurements appropriate?	Y	Y	Y	Y	Y	Y	Y	Y	Y	Y		Y	Y	Y	N	Y	Y	Y	Y
Is the risk of nonresponse bias low?	Y	Y	Y	Y	Y	N	Y	Y	Y	Y		Y	Y	Y	N	Y	Y	Y	Y
Is the statistical analysis appropriate to answer the research question?	Y	Y	Y	Y	Y	Y	Y	Y	Y	Y		Y	Y	Y	Y	Y	Y	Y	Y
**Q5. Mixed Methods Studies**																			
Is there an adequate rationale for using a mixed methods design to address the research question?																			
Are the different components of the study effectively integrated to answer the research question?																			
Are the outputs of the integration of qualitative and quantitative components adequately interpreted?																			
Are divergences and inconsistencies between quantitative and qualitative results adequately addressed?																			
**Quality Score**	80%	80%	100%	100%	100%	60%	100%	100%	100%	100%	60%	100%	100%	100%	60%	100%	100%	100%	100%

Note: #: number, Y: yes, N: no.

## 3. Results

### 3.1. Characteristics of Selected Studies

Table 1 provides a summary of studies included in this review paper. The articles were published between 2011 to 2022. Eighteen studies used descriptive research designs and one study used a quasi-experimental design [23]. The studies were conducted in the Republic of Korea. All studies were quantitative, cross-sectional designs conducted in the Republic of Korea.

Subjects were varied, including students only (n = 5), mothers (n = 11), parents (n = 1), a mother and daughter pair (n = 1), and teachers (n = 1). The sample size of included studies ranged from 101 to 757. Two studies had a sample size of over 700 participants [32,34]. Eleven studies used theory: four used the Health Belief Model [41] and seven studies used the Theory of Planned Behavior [42]. The studies that used a Health Belief Model used variables including perceived susceptibility, seriousness, benefit, and barrier. The studies with the Theory of Planned Behavior used variables of attitude, subjective norm, perceived behavior control. The strengths and limitations of each study are described in Table 1.

From the 19 studies, three themes were identified to be related to the factors associated with HPV vaccination among Korean adolescents: facilitators, barriers, and ways to increase vaccination rates.

#### 3.1.1. Facilitator

Subthemes for facilitators are personal characteristics and environmental factors. The personal characteristics include attitude [12,24,35,36], subjective norms [12,24,28,36], awareness [38], self-efficacy [24,37], perceived benefits [25,31,34], perceived susceptibility [39], perceived barriers [31,39], and perceived behavior control [10,28,30,36], Some papers also mentioned a knowledge aspect to increase intention for HPV vaccination, namely education level [30,38] and high level of knowledge about vaccination [12,23,24,29,34]. Environmental factors were described by various descriptions such as family history of cervical cancer [38], clinician’s recommendation [29,30,39], mothers’ experience of pap-test, concerns about their daughters being high-risk subjects without HPV vaccination, mother’s experience with HPV vaccination [30,35], vaccination status of the health teachers [30] and their children [34], and the child’s teachers’ experience with pap-test [34].

#### 3.1.2. Barriers

Subthemes for the barriers to HPV vaccination included a lack of awareness and knowledge about HPV, concerns about side effects of the HPV vaccine [29,31,33,34,35], such as death, neurological disorders and thrombosis, mothers were reluctant to vaccinate their children, and the burden of HPV vaccine costs [32,33]. Some studies showed that adolescent mothers did not receive the HPV vaccine for their offspring because they did not know exactly what illness the HPV vaccine was related to [24,33,37,38] or did not feel the need to vaccinate [33,38]. Unvaccinated people around adolescents and lack of personal recommendations regarding HPV vaccination were also reasons not to vaccinate [32]. In addition, due to distrust of the effectiveness of vaccination [32,33,34] and concerns about the side effects of the HPV vaccine [33,35,37,38,39], mothers were reluctant to vaccinate their children. Due to a policy that began in 2006, women aged 12–13 are given the HPV vaccine free of charge in South Korea [30], but the fact that the cost of vaccination for other populations is a personal burden [37] has also been shown to be an inhibition of HPV vaccination. Furthermore, addressing these barriers and promoting HPV vaccination requires a comprehensive approach.

#### 3.1.3. Ways to Increase Vaccination Uptake

A total of 12 out of 19 studies reported the relationship between HPV-related knowledge and HPV vaccination [12,23,24,25,26,27,29,33,36,37,38,39] and all papers proposed as recommendation of further actions to increase HPV vaccine uptake by promoting knowledge [10,12,23,24,25,26,27,28,29,30,31,32,33,34,35,36,37,38,39], and coverage of HPV vaccines by public insurance [30,32,33].

### 3.2. Critique of Selected Studies

A total of 11 out of 19 studies were conducted research using a theoretical framework: Health Belief Model [41] and Theory of Planned Behavior [42]. However, there is a limitation that since all of the selected studies were cross-sectional studies, it is hard to reveal a causal relationship and changes over time among knowledge, attitude and intention of HPV vaccination. These two theories are particularly relevant to this review because they provide frameworks for understanding the complex interplay of factors that influence HPV vaccination behaviors among Korean adolescents. By analyzing the factors through these theoretical lenses, the review can offer a more comprehensive understanding of why certain behaviors occur, shedding light on both the motivators and deterrents surrounding HPV vaccination in this specific population.

However, there is a limitation because since all of the selected studies were cross-sectional studies it is hard to reveal a causal relationship and changes over time among knowledge, attitude and intention of HPV vaccination. Therefore, it is necessary to conduct longitudinal studies to identify the change of these variables. Through longitudinal studies, changes in intention over time can be observed, and by analyzing these patterns, potential factors for improving vaccination intention can be identified. Additionally, by tracking the vaccination status of individuals who initially had vaccination intentions, insights can be gained into the actual impact of intention on vaccination behavior. Additionally, eighteen studies were descriptive studies, excluding one quasi-experimental study design [27]. Qualitative research offers the advantage of delving into individuals’ attitudes, beliefs, and experiences in depth, aspects that quantitative research might not fully identify [43]. Therefore, to achieve a comprehensive understanding of HPV vaccination intentions, it’s imperative to incorporate qualitative research, enabling a profound exploration and comprehension.

In addition, there is a limitation in that there was no qualitative study among the selected studies, which might bring a lack of deep understanding of the subject’s experience. There may be a risk of sample bias including recall bias and social desirability bias in that a self-reporting questionnaire was used and convenience sampling was used in limited regional areas in most of the selected studies. For the study subjects, eleven studies were conducted on mothers only, and five studies were conducted on daughters only. In this study, mothers emerged as the primary decision-makers. This is indicative of the Confucian-influenced Korean culture, where mothers traditionally hold a central role as caregivers. However, there is a need to discuss other perspectives that can influence decision-making, such as fathers, relatives, others, and healthcare professionals, reflecting the requirement for a broader discourse. There was only one study conducted on teachers. Therefore, further research includes various subjects that may affect the intention of HPV vaccination in adolescents.

The absence of any mention of controlling for potential confounding factors in the studies is a notable limitation. Addressing potential confounders is crucial, particularly when examining vaccination behaviors and beliefs. This could involve adjusting for variables that might influence the relationship between the studied factors and vaccination outcomes, ensuring more accurate and reliable findings. Therefore, it’s important for future research to consider and account for these confounding factors to enhance the validity of the study results. All investigations were exclusively conducted within the confines of the South Korea. It is imperative to underscore that the idiosyncratic cultural, societal, and policy nuances inherent to South Korea may yield findings that are discernibly disparate from research endeavors carried out in alternative nations. Such context-specific intricacies underscore the prudence required when extrapolating these results to dissimilar cultural or policy contexts.

Within the framework of a systematic review, the observed significant variance in sample sizes (ranging from 101 to 757) does not pose a substantial concern. This is primarily attributed to the nature of systematic reviews, which prioritize the synthesis and interpretation of existing research findings rather than the execution of fresh statistical analyses. Consequently, there is no substantive basis for apprehension when it comes to the comparison or amalgamation of results across these diverse studies within this review’s purview. Nonetheless, it is advisable to remain open to potential avenues for supplementary analyses in the future, aiming to investigate any latent influence that variations in sample size might exert on the outcomes under scrutiny.

## 4. Discussion

**Figure 2:**
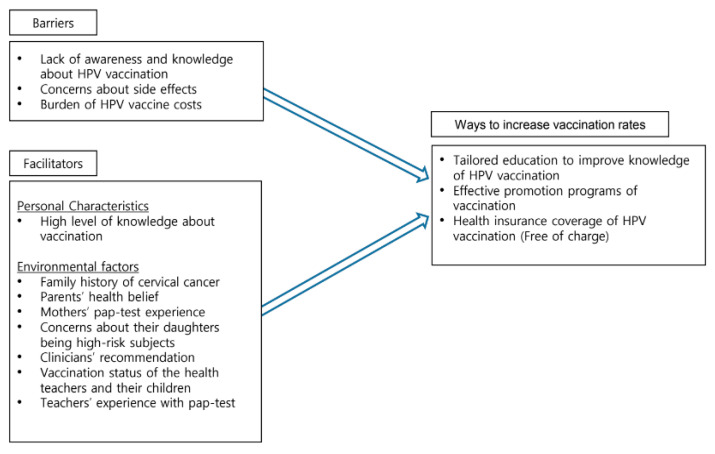
Themes: Vaccination barriers, facilitators and ways to increase vaccination rates.

This study was conducted to identify factors associated with HPV vaccination among Korean teenagers through an integrative review and to provide basic data for education and promotion programs that might increase vaccination rates among Korean teenagers. We reviewed 19 articles to investigate the facilitators and barriers to HPV vaccination among Korean teenagers. Three themes emerged from this integrative review: facilitators of HPV vaccination, barriers to vaccination, and ways to increase vaccination uptake. The facilitators of HPV vaccination uptake include personal characteristics and environmental factors. Individual factors were knowledge about vaccination and self-efficacy and environmental factors were described as family history of cervical cancer, clinician’s recommendation, and mother or teacher’s awareness of the importance of HPV vaccination. The barriers include a lack of awareness and knowledge about HPV of parents including both fathers and mothers, or clinicians, concerns of the side-effects of the vaccination, and the burden of costs. We also provided ways to increase vaccination uptake based on the findings from the integrative literature review. We created a diagram to show the themes as shown in Figure 2. 

### 4.1. Knowledge and Awareness

While the majority of the studies examined in the review emphasized the significance of experience and awareness, it is important to note that the correlation between vaccine-related knowledge and intention to vaccinate remains complex. Despite this weak correlation, possessing sufficient knowledge related to vaccines serves as a reinforcing factor, compared to a lack of knowledge and awareness that becomes a hindrance. Korean adolescents have less knowledge about the HPV than their Western counterparts [27]. The knowledge aspect becomes a good opportunity to enhance the vaccination rate. The level of knowledge can be increased through simple education training, and increased knowledge levels are associated with acceptability and positive perception of the HPV vaccines [44]. Because the decision to vaccinate adolescents with HPV rests with the parents [45], this health education can be given to both adolescents and parents.

There may be gender differences in HPV vaccination between male and female students. In general, the HPV vaccine vaccination and completion rates for male are lower than for female [46,47]. They may be aware of cervical cancer, but they perceive that they are unlikely to be affected by the virus [48] and they tend to have limited knowledge about HPV virus and HPV vaccination [49]. According to a review study targeting male students, some studies reported that the higher the knowledge, the higher the vaccination rate, but other studies reported that this was not the case [50]. Another study reported that recommendations for HPV were associated with higher HPV completion rates in male students [47]. This discrepancy necessitates a continued discussion on the factors that influence the HPV vaccination behavior of boys and girls.

### 4.2. Family and Peer Influences

The fear of side effects becomes a barrier to vaccination rate, as stated in five articles [10,35,37,38,39]. Parents’ low knowledge about vaccines and doubts about their safety are the main reasons for reluctance to vaccinate against HPV [51]. The reason parents are hesitant to vaccinate their children may be due to the side effects of the vaccine itself, but it may also include distrust in policies and safety management [52]. However, this is in contrast with a qualitative study in Australia which found that parents trust the vaccine as it has gone through clinical trials [45]. These different viewpoints highlight the necessity for health authorities and experts to establish effective communication strategies to alleviate parental hesitancy. Specifically, improving knowledge about HPV vaccination including its safety has the potential to play a role in promoting decision-making regarding vaccination.

People around adolescents such as mother, family, teacher, and health professionals are likely to influence the HPV vaccination rate. The mother’s experience with HPV vaccination was a significant predictor of their children’s’ HPV vaccination status. For the mothers of sons, subjective norms, attitudes, and perceived behavioral control were found to be significant predictors of intention to obtain HPV vaccination [28]. A family history or a mother’s experience of a cervical smear may increase the mother’s self-efficacy, leading to an enhancement of the child’s intention to vaccinate [34,38,53]. This is in line with a study showing that increasing parents’ acceptance and intention to receive the HPV vaccine is effective in improving the acceptability of female adolescents to receive the vaccine [54].

However, mothers of elementary school girls often have inaccurate information about the appropriate age for their children’s HPV vaccination [24]. Some parents have a false belief that vaccination will increase adolescent sexual activity, but it is not associated with increased sexual activity [55]. In the systematic review regarding HPV vaccination rate and intention among female, researchers suggest the necessity to measure multiple factors such as risks of vaccination, incorrect information regarding vaccination and vaccination decision-making based on the benefits and effectiveness of vaccination, as oppose to simply measure knowledge of vaccination [13]. Positive beliefs and sufficient knowledge are related with parent’s willingness to vaccinate their adolescents [51]. It is important to highlight that positive beliefs could also play a supporting role, possibly working in combination with sufficient knowledge to increase parents’ willingness to vaccinate their adolescents. Given this, an educational approach is necessary in which health professionals provide parents with accurate knowledge while promoting self-efficacy which was suggested by the two reviewed studies. The self-efficacy was mediated by the relationship between the knowledge of the HPV vaccine and the intention of mothers of female elementary students to receive vaccination [24,37].

### 4.3. School Influences

At the community level, schools are central in accommodating education about HPV including HPV vaccination and screening [56] through teacher and school-based vaccination program. From the reviewed papers, teacher is another party who influences adolescents to get vaccination. Factors associated with the intention to HPV vaccination were associated with the intention to recommend HPV vaccination for girls, the HPV vaccination status of the health teachers of the teenagers, and the teachers’ pap-test experience, perceived benefits, perceived susceptibility, and perceived barriers [34]. In-school education can improve adolescents’ knowledge and understanding about HPV [56]. However, a study showed half of the school staff and parent respondents in a study in French doubted the safety of HPV vaccination. Therefore, it is advised to enhance school staff knowledge about the evidence-based practice of HPV before giving information to adolescents in school [57]. Most of all, it is important to make school staff aware about their potential influence on adolescents’ and parents’ attitudes toward HPV vaccination [58]. School can also contribute to the increase in HPV vaccination rate through school-based vaccination programs. Compared to education only, school-based vaccination and community education resulted in higher vaccination rates [59]. Additionally, randomized controlled trials and systematic review of 15 quasi-experimental studies found that school-based educational interventions were effective in increasing HPV vaccine knowledge, attitudes, and cognition [60]. This program facilitates parents’ decision-making about vaccination [45] because it is advised by the government. According to parents, one of the benefits of this program is that getting vaccinated together with peers at school is more convenient than parents taking them to the clinic [45].

### 4.4. Healthcare Providers’ Role

Besides family and school, healthcare providers play an important role in educating adolescents and their parents. Girls were more willing to receive the HPV vaccination if it was recommended by clinicians or parents than by friends or teachers [29,30,32,39]. When female and male adolescents are aware that HPV vaccine prevents cervical cancer, they are more likely to obtain the HPV vaccination [27]. According to the reviewed studies [10,25], awareness and vaccination rates among male adolescents are low. This necessitates national attention and policy to increase the vaccination rate among this population.

In this review, health care provider recommendation was identified as a reinforcing factor associated with HPV vaccination. This is consistent with research in several other countries [61], which showed that healthcare workers and family members are the most influential sources of information for vaccination. In addition to HPV vaccination, studies on influenza vaccination have shown that medical encouragement has become an important factor [62,63]. This appears to be due to the perception that healthcare providers are trusted professionals who provide more accurate information and advice than friends and family [58].

When a healthcare provider has a higher level of knowledge, they are more likely to recommend vaccines to their patients [64]. Conversely, healthcare professionals’ judgments about whether to recommend a vaccine in a healthcare setting may limit vaccine access to adolescents, regardless their beliefs or preferences [65]. Therefore, it will be helpful if medical staff (doctors and nurses) provide education or campaigns for adolescents focusing on vaccination’s benefits and preventive effects to improve vaccination rates. In randomized controlled trials (RCTs) that provided pediatric healthcare providers with online communication training in three modules including (1) an effective approach to the patients (teenagers), (2) confident recommendation of the vaccination, and (3) talking with parents who hesitate about their teenagers’ vaccination, particularly increased the HPV vaccination initiation (=the first shot of vaccine) [66]. A higher engagement toward videos of narrative-based health information on social media increased the intention to discuss HPV with health professionals. Therefore, it would be an interesting, when promoting HPV vaccination, to promote the parents’ willingness to discuss and get their adolescents vaccinated [67].

### 4.5. Policies and Costs

A further predictor of their daughter’s HPV vaccination was the mother’s health beliefs. The most often occurring predictors were perceived benefits and perceived barriers. In the reviewed papers in this study, the high cost of vaccines is one of the alleged barriers. The vaccination is necessary to receive three times to get the effect but each cost is about KRW 200,000 which is equal to USD 180 and total of three shots is about KRW 600,000 (=USD 540).

A policy is needed to expand the number of free vaccinations [30] from women and adolescents aged 12–13 to men and various ages. This can be seen from the fact that in a previous study [37], 76.2% of adolescent mothers said they would receive the HPV vaccine if it were covered by health insurance. In the UK, HPV is provided to all adolescents aged 12~13 through school, which prevents the issues regarding inequalities or social deprivation in vaccination uptake to be raised. It also contributes to increase the vaccination rates as high in the UK [68,69,70]. The target of the South Korean government support has been expanded to include female youth aged from 12 to 17 years old and low-income women aged 18 to 26 since 2022 [71]). According to an analysis conducted in the US, three interventions for increasing HPV vaccination including reminder of vaccination, school-located vaccination, and the quality improvement of visits to the clinics are cost-effective to prevent 3000 to 14,000 new cases of HPV cancers in the next 50 years [72]. The school-located vaccination in their intervention includes the process of insurance claims for students who use private insurance.

### 4.6. Recommendations for Improvement

HPV vaccine requires a second or third dose depending on age. This means it is important not only to start getting the vaccine but also to complete the second or third dose. Vaccine completeness is known to be significantly lower than the rate of getting first doses [73], and vaccination completion rates for 11- to 26-year-olds are less than the 80% target of the 2020 Healthy People in the United States [74]. In Korea, HPV vaccine completion among females is 65.74% [75]. Addressing this gap necessitates comprehensive strategies. Educating people should aim not only to inform them about the importance of completing ongoing HPV vaccination but also to investigate the underlying reasons for vaccine incompleteness. Efforts will also be needed to identify and resolve misinformation about vaccines, such as concerns or misunderstandings about potential long-term side effects of vaccines.

In Korea, there is a study showing that preventive education based on Planned Behavior theory is applicable to college students [76] and a study that HPV vaccination education affects vaccination-related knowledge, health beliefs, and vaccination intentions for male college students [77]. Since lack of knowledge and awareness are inhibitors to vaccine uptake [24,33,37,38] and are major determinants of vaccination acceptance [61], there is a need to focus on strategies to increase knowledge and awareness of vaccination in the provision of educational interventions. Adults who can influence HPV vaccination decision-making in adolescents can be educated about the vaccine’s effectiveness and importance. In particular, as the recommendation of medical staff and acquaintances of the adolescents has influenced HPV vaccination [10,30], it is considered necessary to provide education and promotion campaigns related to HPV vaccine through health teachers and health professionals. According to a scoping review, encouragement, messages, and phone calls about vaccination using social media can help improve knowledge and awareness of the HPV [78]. Sending individualized reminder messages about following vaccinations via text or email and providing interventions before, during, and after vaccination [79] helps increase vaccination completion rates. In a family practice setting, providing 10-step interventions which included inquiry, posters and brochures, strong recommendations, a presumptive approach, an audit and feedback, performance board, vaccine clinic, staff breakfasts to evaluate and encourage the project, email reminders, and standing orders had a positive effect on improving HPV vaccination rates in adolescent patients aged 18~26 years [79]. In a study of developmentally appropriate and parent-acceptable comic book interventions for East African adolescents in the US, this intervention was able to increase HPV vaccination rates by effectively initiating parent–adolescent discussions about the HPV vaccine [80]. A rigorous multi-step process that integrate theory and focus group help create culturally appropriate guideline for the adolescents [80]. Moreover, a holistic approach to sexual and reproductive health beyond a focus solely on HPV vaccination targeting adolescents will improve adolescents’ overall health behavior and, as a result, increase acceptance of the vaccine. Given the various factors influencing HPV vaccination rates, from individual knowledge levels to healthcare system interventions, an integrated strategy is essential for promoting both HPV vaccination initial uptake and completion.

#### Limitations

Since this review included only published papers, studies with less impressive results may have been excluded. Additionally, the search terms and methods used to finally select papers may have resulted in missing some papers, which may bias our findings and assumptions. Since this study was conducted considering only Korean populations, there are limitations in generalizing the results of this study to HPV-related research, but it may have the advantage of comparing various barriers and facilitators between countries.

## 5. Implications

The three themes of this review’s findings imply the need for nurses to accommodate all of these aspects to increase HPV vaccination in both male and female adolescents. Approaches can be made for individuals, families, communities, health professionals, and policymakers.

### 5.1. Family and Individual Approaches

Individual and family approaches will be very closely related because HPV vaccination in adolescents is likely to be influenced by the knowledge and experience of the mother, as well as the experiences of family members about cervical cancer. Health education can be carried out through various teaching methods and media, and use developmentally appropriate interventions including interactive discussions, informative presentations, role-playing scenarios, informative videos, quizzes, and interactive simulations. When clinicians or educators conduct health education, it is necessary to pay attention to the psychological condition of parents because it involves family values such as the belief that HPV vaccination is related to sexual activity in adolescents. To counteract such misconceptions, public health campaigns aimed at correcting misinformation is crucial.

### 5.2. Community Involvement

Involvement of the community such as school teachers cannot be ruled out as their experience with the pap test and awareness increase the possibility of vaccination in adolescents. Therefore, nurses could assess teachers’ knowledge and experience to better equip them to have a positive influence on parents’ and adolescents’ decision-making regarding HPV vaccination. It is also important to encourage schools to participate in the promotion of HPV vaccination by incorporating information about cancer and HPV vaccination into the curriculum and by becoming HPV vaccination sites through school nurses. This adjustment could also involve integrating comprehensive sexual health modules into the curriculum that provide students with information about the significance of HPV virus and HPV vaccination. By doing so, students can become informed about safeguarding their health and making informed decisions regarding preventive measures. Additionally, workshops and interactive sessions could be organized to address questions, concerns, and misconceptions surrounding HPV vaccination, fostering an environment of open dialogue and knowledge dissemination. This approach would empower students with the necessary information and encourage proactive engagement with their sexual health and well-being.

### 5.3. Health Professionals Roles

Health professionals have a crucial role in imparting health education to individuals, families, and communities. They provide essential information and guidance on various aspects of well-being, such as disease prevention, healthy lifestyles, proper nutrition, and fitness. This empowers people to make informed decisions about their health. Health professionals should first be provided with sufficient information about HPV vaccination and techniques to approach adolescents, families, and communities when educating them. This improves their ability to understand the reasons why some adolescents remain unvaccinated and determine appropriate education methods for adolescents and families.

As an example of this role, health professionals can employ tailored reminder messages and digital platforms to enhance vaccination adherence. Tailored reminder messages can play a pivotal role in prompting individuals to adhere to their vaccination schedules. However, discussing the broader aspects of follow-up, such as establishing structured communication channels between healthcare providers and vaccine recipients, as well as leveraging digital platforms for appointment scheduling and tracking, would underscore the importance of seamless vaccination continuity.

Recognizing potential limitations and challenges in implementing these interventions strengthens their essential role in comprehensive vaccination strategy. These could include factors like vaccine hesitancy, accessibility to healthcare facilities, and cultural barriers. Addressing these challenges might involve community engagement efforts to dispel misinformation, mobile vaccination clinics to reach underserved populations, and culturally sensitive educational materials. Acknowledging both the benefits and potential difficulties can provide a more balanced assessment of the proposed interventions’ feasibility and impact.

### 5.4. Policymakers

Nurses can also advocate for policy changes regarding free vaccination expansion of the age and gender coverage through greater insurance coverage for HPV vaccination for female and male adolescents. An analysis of the cost-effectiveness of HPV vaccination can be carried out by comparing the costs of vaccination interventions and future cases of HPV to show a compelling reason to increase insurance coverage. Nurses are central to advocating for policies that could potentially boost the HPV vaccination uptake.

### 5.5. Actional Steps

Furthermore, we suggest the need for specific and actionable interventions to enhance HPV vaccination rates through efforts that go beyond knowledge and awareness, incorporating a more behavioral dimension. This includes implementing educational workshops, online modules, or community outreach programs. The growing influence of digital media in health education is not adequately addressed. Mobile apps, text reminders, or interactive online platforms could be considered as tools for education and reminders. Furthermore, it is essential to collaborate with the media to dispel misconceptions about the HPV vaccine and promote the importance of vaccination.

In addition, it is necessary to consider how the success of the proposed interventions will be measured. For example, it should explore not only an increase in vaccination rates but also intermediate metrics like increased awareness, positive attitudes towards vaccination, and improvements in adolescent sexual health knowledge. Given the sensitive nature of topics like sexual health in certain cultures, these strategies could benefit from further emphasizing the importance of culturally sensitive approaches. In addition to the broader cost-effectiveness analysis of HPV vaccination, the review could suggest smaller economic incentives, such as discounts or vouchers, to encourage vaccination.

## 6. Conclusions

Preventive HPV vaccination in adolescent boys and girls has the potential to significantly reduce a variety of HPV-related morbidity and mortality. This study was conducted to identify the factors that influence female and male HPV vaccination intention considering personal, parental psychosocial, and environmental factors. After reviewing the studies, barriers and facilitators were identified. The findings highlight the importance of an approach that considers individual, family, community, and policymaker aspects to increase HPV vaccination coverage.

### Recommendations

In order to increase the vaccination uptake, various techniques, such as educational events and curriculum adjustments, should be implemented. Moreover, because clinicians’ recommendations are a facilitator of parents’ decisions to vaccinate their children, involving both male and female adolescents, parents, as well as clinicians in school-based immunization programs or educational events may contribute to increasing vaccine immunization rates. Since economic issues are a major consideration for HPV vaccination, further expansion of vaccination cost support for economically vulnerable groups should be included, and customized notification messages and educational interventions to complete the entire vaccination process will be recommended.

The studies analyzed for the review did not include qualitative research, the study subjects were mostly female adolescents, and there was a lack of research targeting medical providers. Future research to promote HPV vaccination will require an in-depth understanding of the subjects through qualitative research. Additionally, a review of papers targeting only male adolescents would be meaningful, and it is necessary to conduct research on medical staff to deepen our understanding of them.

## Data Availability

This is the research based on published literatures. The data are collected in these literatures. The data presented in this study are available in references [10,12,23,24,25,26,27,28,29,30,31,32,33,34,35,36,37,38,39].
